# ‘Staying strong on the inside and outside’ to keep walking and moving around: Perspectives from Aboriginal people with Machado Joseph Disease and their families from the Groote Eylandt Archipelago, Australia

**DOI:** 10.1371/journal.pone.0212953

**Published:** 2019-03-11

**Authors:** Jennifer J. Carr, Joyce Lalara, Gayangwa Lalara, Gloria O’Hare, Libby Massey, Nick Kenny, Kate E. Pope, Alan R. Clough, Anne Lowell, Ruth N. Barker

**Affiliations:** 1 College of Healthcare Sciences, James Cook University, Cairns, Queensland, Australia; 2 Machado Joseph Disease Foundation, Alyangula, Northern Territory, Australia; 3 Community-based Health Promotion and Prevention Studies Group, College of Public Health, Medical and Veterinary Sciences and Australian Institute of Tropical Health and Medicine, James Cook University Cairns, Australia; 4 Northern Institute, Charles Darwin University, Darwin, Northern Territory, Australia; Western Sydney University, AUSTRALIA

## Abstract

Machado Joseph Disease (MJD) (spinocerebellar ataxia 3) is a hereditary neurodegenerative disease causing progressive ataxia and loss of mobility. It is the most common spinocerebellar ataxia worldwide. Among Aboriginal families of Groote Eylandt and related communities across Australia’s Top End, MJD is estimated to be more prevalent than anywhere else in the world. This study explored lived experiences of individuals and families with MJD to determine what is important and what works best to keep walking and moving around. A collaborative qualitative exploratory study, drawing from constructivist grounded theory methods, was undertaken for data collection and analysis. Semi-structured in-depth interviews were conducted with individuals with MJD (n = 8) and their family members (n = 4) from the Groote Eylandt Archipelago where ~1500 Aboriginal people (Warnumamalya) live. Interviews were led by Warnumamalya community research partners in participants’ preferred language(s). Participants described their experience of living with MJD, from ‘knowing about MJD’, ‘protecting yourself from MJD’ and ‘adjusting to life with MJD’. While the specific importance of walking and moving around differed widely between participants, all perceived that walking and moving around enabled them to do what mattered most to them in life. ‘Staying strong on the inside and outside’ (physically, mentally, emotionally, spiritually) was perceived to work best to keep walking and moving around as long as possible. A framework that included personal and environmental strategies for staying strong emerged: ‘Exercising your body’, ‘having something important to do’, ‘keeping yourself happy’, ‘searching for good medicine’, ‘families helping each other’ and ‘going country’. This study, the first to explore lived experiences of MJD in Australia, highlights the importance of maintaining mobility as long as possible. Strategies perceived to work best address physical and psychosocial needs in an integrated manner. Services supporting families with MJD need flexibility to provide individualised, responsive and holistic care.

## Introduction

The Aboriginal people of the Groote Eylandt Archipelago (Warnumamalya) [1] in Australia have experienced the impact of Machado Joseph Disease (MJD) on their families for generations [2]. MJD, or spinocerebellar ataxia 3 (SCA3) [3], is an autosomal dominant neurodegenerative disease estimated to be more prevalent in affected Aboriginal communities in the Top End (northern region) of Australia than anywhere else in the world (Groote Eylandt Archipelago ~743/100,000: Azores Archipelago ~39/100,000) [4–7]. Each child of a parent with MJD has a 50% chance of developing the disease and at an earlier age due to genetic anticipation [8]. In some instances, three generations of individuals in one family can be affected at any one time [6]. Progressive decline in mobility due to ataxia is a characteristic feature of the disease with most individuals wheelchair bound within 10–15 years of symptoms emerging. In the absence of pharmacological interventions to alter the progression of MJD and a mean life expectancy of 20 years from onset of symptoms, loss of mobility places a substantial disability burden on individuals and families with MJD [9].

MJD is estimated to have been present in families from the Groote Eylandt Archipelago for more than 7000 years [3], yet was not diagnosed until the 1990s, when the gene mutation was first confirmed [10]. By the late 1990’s and early 2000’s, individuals with MJD were dying earlier than in previous decades and increasing numbers of Aboriginal people in other communities across the Top End were identified with the disease. The grief and hardship experienced by families with MJD in these remote Aboriginal communities was, and continues to be, compounded by significantly higher rates of disability and chronic disease compared to non-Aboriginal Australians [11]. Limited access to health services, carers, and accessible housing, as well as poor infrastructure and overcrowding, marginalisation and isolation from mainstream society are some of the issues faced [9, 12]. Enormous demands are placed on families, so much so that many individuals are often forced to move hundreds of kilometres away from their families and communities for care.

Testament to the strength and resilience of families of the Groote Eylandt Archipelago, in 2008, Warnumamalya came together with trusted local community members to establish the MJD Foundation (MJDF). The purpose of the MJDF was to provide comprehensive services and engage in research to support families with MJD [13]. One area of concern for families was the decline in mobility, which had not been previously explored within the unique cultural and geographical context of Aboriginal communities affected by MJD. Recognising that services and supports provided for Aboriginal people risk failure if they do not take into account their views, lifestyle and concepts with respect to cultural and traditional practices [14], the aim of this study was to explore, from the perspective of individuals and families with MJD of the Groote Eylandt Archipelago, (1) ‘what is important’ and (2) ‘what works best’ to keep people with MJD walking and moving around.

## Methods

### Study design

A collaborative qualitative exploratory design drawing on constructivist grounded theory methods [15, 16] was undertaken. To privilege the voices of Aboriginal families with MJD as experts living with the disease [17, 18], this study drew from Indigenous and Participatory methodologies. The design ensured the research process was responsive to families, community research partners (CRPs) and cultural protocols [15, 17]. The research team consisted of Warnumamalya CRPs (authors JL, GL, GO), who were experienced community workers and cultural advisors, and either individuals with MJD or a close family member of an individual with MJD. A non-Aboriginal researcher (JC) who was an experienced physiotherapist in neurological rehabilitation, was invited to design and undertake the research (supported by RB). Non-Aboriginal associate investigators (RB, AL and AC) supervised the research and shared more than 20 years of experience working and conducting research in remote Aboriginal communities in the Top End of the Northern Territory (NT). Ethics approval was granted prior to study commencement by the Human Research Ethics Committee (HREC) of the Northern Territory (NT) Department of Health and Menzies School of Health Research (HREC 2016–2672) and James Cook University HREC (H6860). Permission to conduct this research was granted by the Anindilyakwa Land Council (ALC).

### Setting

**Fig 1:**
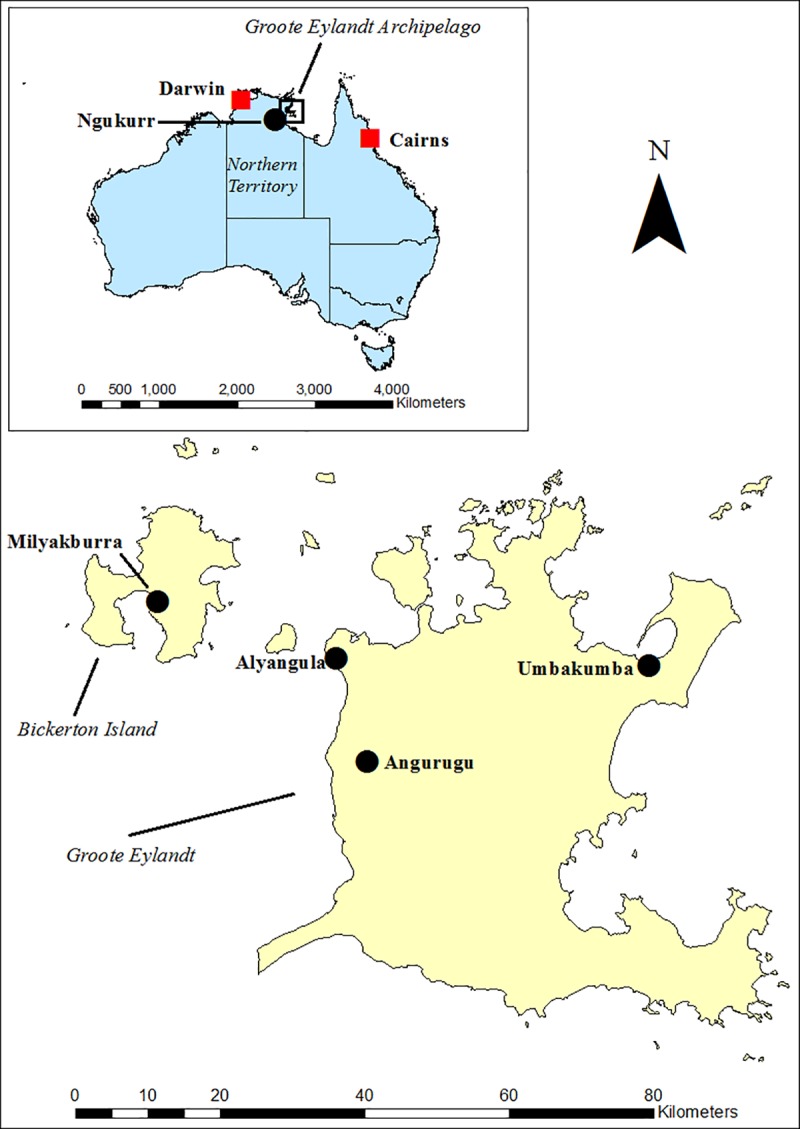
Localities of participants: Groote Eylandt Archipelago (Angurugu, Umbakumba, Alyangula, Milyakaburra) and Ngukurr on mainland Australia. Map created with ESRI ArcGIS using Australian Bureau of Statistics (ABS) data [20]. Red, major cities; black, Aboriginal communities.

This study was conducted with individuals and families with MJD who identified as belonging to Aboriginal communities of the Groote Eylandt Archipelago (Angurugu (population ~ 855), Umbakumba (population ~ 503) and Amakalyakba (Bickerton Island) (population ~ 137) [7], including those who had relocated (Fig 1). Warnumamalya are the Traditional Owners of the Groote Eylandt Archipelago and have occupied the area for up to 8000 years [19].

Groote Eylandt, named in 1624 by Dutch explorers, is the third largest island in Australia, situated off the east coast of Arnhem land in the Gulf of Carpentaria [21]. The closest major city is Darwin (~ 650km away), accessible by plane or barge. Milyakaburra is situated on neighbouring Bickerton Island, accessible by plane or ferry. Each community has a school, supermarket, health clinic, emergency services post, aged care services (day assistance available only), arts services, as well as a lingustics centre in Angurugu [22]. The township of Alyangula is also situated on Groote Eylandt and was established as a residence for employees of the manganese mine, largely home to mining staff and health and education professionals servicing Groote Eylandt [22].

Although rapid cultural change has occurred due to non-Aboriginal influence since the mid 1900s, traditional cultural practices such as ceremonies, hunting and gathering and systems of social organisation remain an important part of life for most Warnumamalya today [22]. Anindilyakwa is the first and main language spoken by Warnumamalya, with English predominantly used to accommodate non-Anindilyakwa speaking visitors [23]. Warnumamalya have been supported by the MJDF since 2008 with physiotherapy services available on Groote Eylandt from 2012.

### Participants

Individuals with a clinical or genetic diagnosis of MJD living in the locations of Groote Eylandt (n = 6), Ngukurr (n = 1) and Darwin (n = 1) (between February 2017 to November 2017) participated in semi-structured in-depth interviews. Family members without MJD were invited to participate at the discretion of the individual with MJD (n = 4). Participants were purposively selected to ensure a wide range of experiences and opinions until theoretical sufficiency was reached, where no further data added dimensions or properties to the emergent categories [24]. Individuals known to the MJDF services were initially approached by MJDF service providers and invited to participate. Interested participants were approached by CRPs and JC to engage in discussion about the study and to seek informed consent. No participants declined to participate or dropped out of the study. All participants were aged over 18 and provided written informed consent prior to the interviews. Participants with a moderate to severe intellectual or psychological disability or impairment were not interviewed to avoid inappropriate participant burden and potential challenges in ensuring informed consent.

### Data collection

Interviews were led by CRPs in collaboration with JC. Interviews were recorded on video, digital audio and/or through note taking, according to the participant’s preference. Participants had the opportunity to select the interview location, communicate in their preferred language, and to participate in further interviews as many times as they felt appropriate to share their experiences fully. In all, participants were interviewed on two to four occasions, and the duration of interviews ranged from 30 minutes to three hours. Interviews followed a culturally congruent conversational style that allowed rich descriptions of experiences, prolonged pauses, and time to build trust and rapport with JC. Questions that guided the interviews are in Table 1. Each participant was invited to draw on their experiences of living with MJD personally and on their experience of supporting their family members with MJD. CRPs collaborated with JC to prepare questions and to translate and transcribe interviews into English. Observations and reflections recorded in field notes were also included as data.

**Table 1:** Interview topic guide.

1. Tell us your story and your family’s story about MJD
2. What is important and what matters most about walking and moving around?
*Probes included*:
*What matters most to you about walking and moving around*?
*Why is walking and moving around and staying strong important to you*?
*What keeps you walking and moving around*?
3. What makes walking and moving around easier or harder?
*Probes included*:
*What gets in the way of being able to walk and move around for you*?
*What things make it hard/tricky to walk and move around with your family*?
*How do these things affect you and your family*?
4. What makes walking and moving around better or worse with MJD?
*Probes included*:
*What helps keep you walking and moving around and staying strong*?
*What makes your walking and moving worse or weak*?
5. What works best to keep people with MJD walking and moving around?
*Probes included*:
*What have you found helps keep you and your family walking and moving around*?

### Data analysis

CRPs and JC worked collaboratively to inductively analyse and interpret the data [16]. NVivo software (QSR International’s NVivo 11) was used to assist with initial and focused coding of transcripts and field notes to identify emergent categories [16]. Regular oral analytical discussions between the researchers occurred to identify, discuss and verify codes from the data and organise them into categories [15]. Illustrations emerged naturally during the discussions to interpret the data and describe emergent categories. Member checking was completed with each participant to confirm and extend the findings and review the fit of categories to their experiences. Following this, additions and modifications were made, and each participant contributed to designing refinements of the final visual representation of the key findings. This visual illustration was used to share and confirm the findings with family representatives and community Elders. Participants were also consulted to gain their views on the implications of the study findings for people with MJD and service providers.

To remain reflexive and avoid misinterpretation of the data, JC collaborated closely with CRPs and families with MJD to facilitate meaning-based translations of participants’ stories. The aim of meaning-based translation, rather than word-for-word interpretation, is to ensure the meaning of the source language (Anindilyakwa) is retained when translated into the target language (English) [25]. Review of the literature prior to study commencement was limited to minimise the influence of preconceived ideas on interpretations of the data [26]. Following established procedures [27], peer review of the data throughout analysis was undertaken by an experienced qualitative researcher (RB).

## Results

Characteristics of study participants are outlined in Table 2. Living with MJD shaped participants’ perspectives on walking and moving around. Mirroring the progressive onset of symptoms, three phases emerged with participants sharing their stories about living with MJD: from ‘knowing about MJD’, ‘protecting yourself from MJD’, and ‘adjusting to life with MJD’. Walking and moving around was important for a range of different reasons, yet essentially enabled participants to do what mattered most to them in life. For what works best to keep walking and moving around, participants emphasised ‘staying strong on the inside and outside’ by ‘exercising your body’, ‘having something important to do’, ‘keeping yourself happy’, ‘searching for good medicine’, ‘families helping each other’ and ‘going country’. In the following, for each of these strategies in turn, selected evidence provided by Warnumamalya is presented and interpreted.

**Table 2:** Characteristics of study participants.

*Participant Characteristics*	*Number (%)*
*Individuals with MJD*	8
*Age range (yrs)*	
30–39	3 (37)
40–49	5 (63)
*Age of onset (yrs)*	
20–29	2 (25)
30–39	5 (63)
40–49	1 (12)
*Gender*	
Female	5 (63)
Male	3 (37)
*Lives Alone*	
Yes	0 (0)
No	8 (100)
*Partner*	
Yes	6 (75)
No	2 (25)
*Employed*	
Yes	2 (25)
No	6 (75)
*Mobility status*	
Independent	3 (37)
Requires assistance*	3 (37)
Wheelchair dependent	2 (26)
*Activities of daily living*	
Independent	3 (37)
Requires assistance**	5 (63)
*Family members of those with MJD*	4
*Age*	
30–39	2 (50)
40–49	1 (25)
>50	1 (25)
*Gender*	
Female	3 (75)
Male	1 (25)

*, Requires physical assistance and/or mobility aid for indoor/outdoor mobility

**Requires physical assistance or assistive devices.

### Knowing about MJD

Before the ‘sickness’ was identified as MJD, participants recalled the shame, sadness and worry it brought to the family. They spoke of wanting to hide their disease from the community and not talk about it. At the same time, they were watching their parents with the disease, noticing symptoms in themselves, then seeing signs of the disease in their own children, nieces and nephews.

“*…when his mother was in a wheelchair*, *he would help lift her up every day and every night…he was making himself strong*.*… then when she passed away… he knew then maybe that that sickness would jump to him*.*”* (10)

Once identified, families began understanding MJD and losing feelings of shame, realising no one was to blame. Yet, knowing did not stop feelings of grief and sadness for the loss of family members, loss of their own independence, worrying about the burden they would place on family and worrying for those in the family yet to find out they too had MJD.

“*Yeah*, *I’m worried about…the girls when they clean up my house… and I been get this sickness… I want to be with those girls to help them*. *And I want to work*, *like some of the strong people*, *they work*. *I want to be like them*. *I want to work like them…” (3)*

Family members without MJD reported struggling with the knowledge that some in the family have MJD while others do not, wishing the disease on themselves rather than watching family suffer. They recognised that the support provided by the MJDF for individuals and families today, was far greater than 30 years ago, reducing the burden on their family, promoting awareness and reducing stigma. They felt proud that the impact of MJD was being recognised and that ‘black and white’ were working together, to support families with MJD to live a good life.

“*Back then*, *there was no one*, *only me*. *Now I sit here today*, *and I am proud*.*” (11)*

### Protecting yourself from MJD

Rather than dwelling on their sadness, participants reported focusing on living a happy life as long as possible. Finding ways to avoid or delay the impact of MJD offered a pro-active way of protecting themselves and their families from the disease. For some, this meant hiding away, not engaging with family or services or ignoring associated medical problems. Others preferred not to talk about the disease, fearing being treated differently. Family members without the disease also found MJD difficult to talk about, preferring instead to talk about how they were helping their family to live a happy life.

When finding out they had MJD, some participants immediately wanted to do something about it, to protect themselves and prepare for the journey ahead. They became more physically active, searching for activities to help themselves and their families, believing that keeping the body moving or ‘keeping the blood pumping’ ‘stops the disease from covering you’, and can ‘protect you from MJD getting worse’. Some acted early, before symptoms became apparent while others waited until forced to act, once walking had become difficult.

“So, *when I got that news that I got that MJD in my genes*, *I been thinking nah I gotta do something you know*. *So*, *I started playing sport again*, *playing footy*, *and doing more work… like doing my gardening things*, *like landscaping*, *like digging*, *raking*, *lawn mowing*, *putting in new plants you know*.*” (12)*

Participants stressed the importance of ensuring young people learned about MJD, so they could have time to prepare for the onset of disease and for a meaningful future.

### Adjusting to life with MJD

Participants spoke about adjusting their lives once walking and moving around and participating in work and family roles were becoming challenging.

*“I’m thinking*, *I’m frightened about kids and tripping*, *because I might make mistake*, *I might fall over with the baby*, *kids or people*. *I might*, *you know*.*” (6)*

Adjusting to life with MJD would often mean using mobility aids and accepting help from others. Adjusting could also mean managing consequences of the disease, such as bladder and bowel dysfunction, fatigue, cramps, falls, pain, and other illnesses such as colds, flu and infections. Nevertheless, participants found ways of adjusting, ways of pushing themselves to move around the community to do what was important to them, calling on support or trying new ways of doing things.

### What is important about walking and moving around?

Being able to keep walking and moving around was perceived as extremely important by all participants because it enables individuals and families with MJD to do what matters most to them in their lives. What ‘mattered most’ varied widely between participants and included caring for family, working, participating in ceremony, hunting, fishing and contributing to the community.

“*Important things are like*, *going out bush*, *hunting and making spears*, *doing traditional dancing*, *they are the main things for me that are important… If I couldn’t walk*, *I wouldn’t be able to do any of those things you know*.*” (5)*

### What works best to keep walking and moving around?

When considering what works best to continue walking and moving around as long as possible, participants emphasised the importance of *‘staying strong on the inside and outside’*. Doing so was helping families protect themselves as the disease progressed, enabling them to manage challenges along the way. Participants explained the importance of ‘leaving it up to the person’ to decide what is important to them in life and what strategies will work best in helping them stay strong on the inside and outside.

#### Staying strong on the inside and outside

Staying strong on the ‘outside’ was described as staying physically strong and able, while staying strong on the ‘inside’ was about staying strong emotionally, mentally and spiritually. Emphasis was placed on the fact that the two sides of ‘staying strong’ must be working together to be ‘strong and happy’ throughout life.

“*It’s my life you know…My person*, *my life*. *I like doing this because it’s my own life*, *my own personality*. *Because maybe I’m that kind of person*. *And because I love doing all those things*.*” (5)*

**Fig 2:**
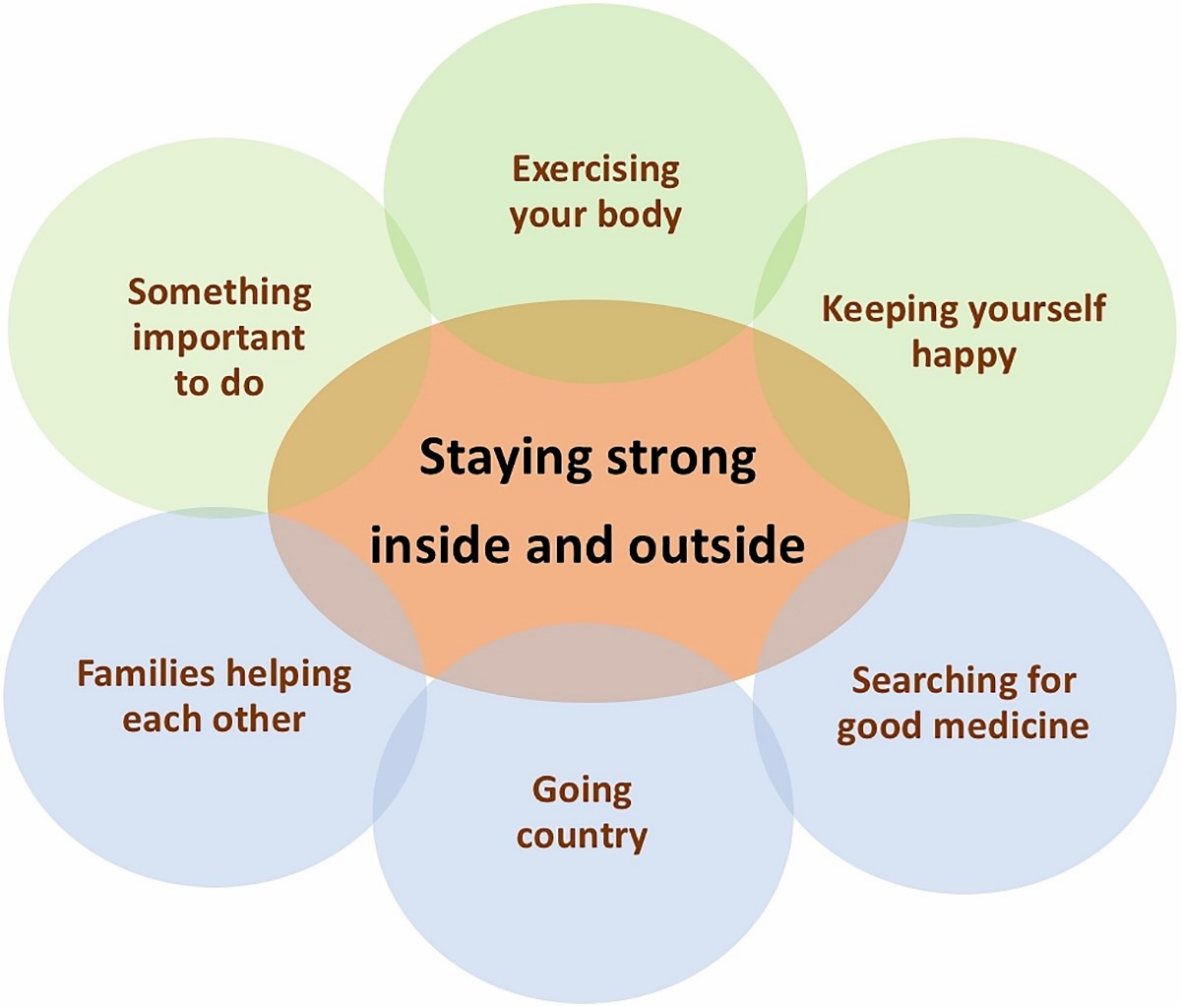
Framework for staying strong on the inside and outside to keep walking and moving around.

A framework outlining strategies or lifestyle practices for ‘staying strong on the inside and outside’ emerged (Fig 2). The framework includes both personal (‘exercising your body’, ‘having something important to do’, ‘keeping yourself happy’) and environmental factors (‘families helping each other’, ‘searching for good medicine’, ‘going country’) that exist within the context of each individual and family with MJD.

**1. Exercising your body.** ‘Exercising your body’ was primarily viewed as being achieved through having an active lifestyle (i.e. going country, fishing, hunting, swimming, dancing, looking after children, cleaning, doing jobs around the home, walking to the shops or to see family). Riding the exercise bike, walking on a treadmill or doing leg and arm strength training was considered a way of complementing an active lifestyle. Keeping the body moving every day, with more strenuous exercise at least once a week, was recommended. When seeing their parents’ generation, living longer lives with MJD compared to their own generation, participants attributed their parents longevity, in part, to the active lives they had led by being hunters, without vehicles.

“*Keeping the body and the blood moving stops the disease coming quickly*.*” (7)*

Participants felt that sitting or sleeping too much caused faster disease progression. While a number of participants felt they liked exercising together in pairs or a group for mutual support, others preferred exercising in a private place, not ‘in front of an audience’ or with people they must avoid according to traditional custom. Sadness was expressed about others where shame or embarrassment was preventing them from exercising.

“*And it doesn’t matter about*, *they need to forget about shame things*, *they can get rid of that*, *and just go for it*, *make themselves strong and do exercise*.*” (2)*

‘Exercising your body’ was thought to be helping with worrying and sadness, regardless of level of mobility, perhaps reminding individuals about when they were living *without* MJD, helping them to feel happy and positive. Family members reported feeling proud seeing their loved ones exercising and working hard to keep walking and moving around.

To stay active as the disease progressed, participants found ways of making walking and moving around easier. This included taking your time, moving in cooler parts of the day, eating and sleeping well as well as using assistive devices, transport and home modifications. Those who are wheelchair bound were trying to keep exercising in sitting or seeking more physical help to move. With increasing severity of disability, exercising your body could mean relying on support from families and services to keep moving around the community.

**2. Something important to do.** Participants felt that having something important to do was forcing those with MJD to keep moving and to stay physically strong. Having something important to do offered a way to contribute meaningfully to family and community, and to keep feeling strong on the inside. Something important could mean a paid job, hobbies, playing sport, studying, being a parent, or keeping up with caring for yourself and your home to support your family. Participants advocated that those at risk of having MJD, especially children, should get an education, find a job or something important to do for their future, to make sure they have a reason to push themselves to keep walking and moving around.

“*Because if you have to work you have to walk*. *You have to get up and prepare for work*. *You have to get ready for work… You have to prepare for everything you know*. *Like if you are going out there (pointing out at the community)*, *you have to get moving to get there*.*” (5)*

Being unable to work due to progression of the disease caused some participants to worry about getting worse faster. Participants felt that they gained inner strength by pushing themselves and setting an example for their family and others. Having a family that ‘gives you room’ to push yourself to do important things was highly regarded.

“*I push myself*. *So I can show my kids or families… and they can respect me…love me…so when after*, *when I pass away from this disease…When this disease gets worse and worse and worser than me…they can talk to people about this sickness*.*” (6)*

**3. Keeping yourself happy.** Feeling happy and positive was a way participants felt they could keep persevering in life despite having MJD and how families could continue supporting them. Feeling depressed and worried could provoke feelings of physical weakness and reluctance to move around. Strategies for keeping yourself happy, included getting out of the house or community, having time ‘away from the sickness’, going to respite in the city, and taking trips away to see family. Exercising your body, relaxing, having fun, listening to music, doing or watching dancing and managing health issues were also considered to be beneficial. Some felt they were naturally more positive than others, making it easier for them to stay strong while others might be requiring extra support from family and the ‘extended family’ of service providers (i.e. MJDF, Aged Care services, and the health clinic). For those not ready to accept help, continuing to offer them support was important so the person could feel happy in knowing help was there if they needed it.

**4. Searching for good medicine.** ‘Searching for good medicine’, such as traditional bush medicines, bush tucker, and some medicines from the clinic, is seen as part of staying strong on the inside and outside. A belief is shared by many participants that a cure for MJD is undiscovered somewhere in the country around them. Searching for the cure, for some, was a great motivator for keeping walking and moving around. Many traditional medicines were also being used to manage fatigue, improve physical strength and manage other health problems either arising from MJD and/or compromising function (i.e. colds, flu, pain and bladder infections). Medicines from the clinic could also help and were often used together with bush medicine.

Searching for bush medicine is a highly valued activity that brings families together and is viewed as helping to ‘heal you on the inside’. As high levels of mobility are required, gathering bush medicine for those no longer physically able provided ways for families to support those experiencing disability resulting from MJD, who in turn, felt supported by family.

**5. Families helping each other.** ‘Families helping each other’ plays an important part in staying strong. Family support is provided in different forms, varying according to the individual, the family and the stage of MJD. For some, physical assistance from family to move around the home safely, was helping them stay strong on the outside. For others, having families visit and stay with them was offering social support, helping them feel strong inside.

All participants recognised the importance of having help from family, from their own experiences of caring for other family members with MJD. Family members undertook carer roles without need for discussion, just knowing to be there at the right time. Acknowledging that the progression of MJD placed significant demands on families, support from the extended family of service providers was appreciated, alleviating the load and helping families to remain ‘happy and strong’. Individuals lacking family support drew heavily on these services.

**6. Going country.** ‘Going country’ is an integral part of life for Warnumamalya that was considered essential for helping families with MJD to stay strong. ‘Country’ is part of each person’s identity. The term refers to the lands where there is a shared traditional and spiritual connection through stories and knowledge from ancestors, and the people, animals, plants, earth, rocks, water, air, songlines and dreamings of these lands. ‘Going country’ involves extended four-wheel drive travel, walking long distances over uneven ground, sand, through water and mud to go fishing, hunting, visiting special lands, to participate in ceremony and to talk to the ancestors. Going country meant getting out and about, to places meaningful to the individual, to do things things that matter, with people that matter.

*“…Going* bush, *going country*. *Going out looking for bush medicine*. *Moving around*. *I know that it is really important to me and those people with MJD*. *They should keep going out to the bush*. *Get those yams*. *Get those sugar bags*, *fishing*. *It is very important for us*, *with this disease…Because being on country you know… it helps us feel stronger…it makes me feel strong*, *inside it makes me strong*.*” (12)*

While going country was a way of exercising your body, participants acknowledged difficulties ‘going country’ as walking declined or if you were using a wheelchair. For example, difficulties with toileting led to people feeling like a burden or embarrassed if reliant on others. Supporting family to go country could be hard due to problems accessing a vehicle, time spent fulfilling responsibilities to other family members or accessing services in the city. Yet continuing to go country for enjoyment, to ‘feel the breeze’ and to be with family, whether walking or in a wheelchair, helped families stay strong inside amidst the emotional impact of declining physical abilities. All felt emotionally low when unable to go country.

The aforementioned strategies or lifestyle practices reported by participants contribute to the framework for staying strong on the inside and outside, and were perceived to provide individuals and families with MJD the best chance to keep walking and moving around as long as possible. Despite the challenges of the disease, no one was willing to let it overcome them.

*“I want to stay strong you know… I’m going to stay strong…I won’t let it win*.*” (6)*

## Discussion

This is the first study to explore perspectives of individuals and families with MJD, on walking and moving around. The experience of MJD is described as the process of first knowing about MJD, then protecting themselves from MJD and finally adjusting to life with MJD. Walking and moving around were considered essential to do what matters most in life. To keep walking and moving around, regardless of level of disability, staying strong on the inside and outside was thought to work best. While recognising the need to ‘leave it up to the person’ to choose, a range of strategies or lifestyle practices specific to the individual, the family and community context could be employed. Strategies identified included ‘exercising your body’, ‘having something important to do’, ‘keeping yourself happy’, ‘searching for good medicine’, ‘families helping each other’ and ‘going country’. It is proposed that these strategies provide a framework for staying strong that can inform service delivery.

### Supporting individuals and families throughout each phase of MJD

The impact of MJD as described in this study, includes knowing and learning about the disease, long before symptoms are recognised, or a positive genetic test recorded. Participants described how the family is continually living with the impact of MJD over the generations, similar to the experience of those with other hereditary neurodegenerative diseases [28]. Yet, rehabilitation services for this population have typically focused on the individual, rather than families, and commence after onset of symptoms [29–31] when adjusting to life with the disease may be the only alternative [28, 32, 33]. The experience of MJD described in this study aligns closely with the biopsychosocial Family System Genetic Illness (FSGI) model [34], which recognises the ongoing impact on individuals and families along the life cycle and phases of genetic disease [35, 36]. Services designed to support individuals with MJD therefore, need to provide appropriate and ongoing access to information and support. Opportunities for participation in rehabilitation for both individuals and families with MJD are also necessary to minimise the impact of MJD on their lives.

### Leaving it up to the person to decide

The need to ‘leave it up to the person’ was expressed strongly in this current study, in relation to what is important, what works best and how to push oneself to keep walking and moving around. Individual autonomy is of central importance across many cultures [37], with strong links to health and well-being [38, 39]. For Aboriginal and Torres Strait Islander people in Australia, lack of respect for autonomy has led to resistance to external control and avoidance [40]. Hence, culturally-safe, person-centred care [41, 42] that enables individuals and families to make informed decisions [39, 43] is essential. *What will work best* needs to be decided collaboratively with individuals and families and not simply imposed by service providers because ‘it is in their best interests’ [41].

### Responding holistically to both physical and psychosocial needs

Staying strong on the inside and outside means choosing strategies that *simultaneously* address both physical and psychosocial well-being. While this aligns with holistic conceptualisations of health fundamental to Aboriginal and Torres Strait Islander people [44], it is also consistent with the views of other individuals with ataxias, who have advocated for person-centred interventions for physical and psychosocial well-being [45, 46]. The International Classification of Functioning, Disability and Health (ICF) provides a holistic framework to guide assessment and planning to ensure services for families with MJD [47] are inclusive of evidence-informed physical [48, 49] and psychosocial strategies to promote mobility.

### Drawing on contextual strengths–individuals, families and communities with MJD

In this current study, the framework for staying strong on the inside and outside, as described in the results, is reflective of a strength-based approach: both personal and environmental strengths relevant to the context are drawn upon to find solutions. Such strengths extend across capacity, skills, knowledge and connections of the individual, family and community [50]. Despite differences in culture, lifestyle and geographical location, such an approach has relevance to individuals and families with MJD or other neurodegenerative diseases found around the world. Listening and learning from each individual and family about the strengths which exist within their context provides the foundation for collaboration between service providers and families to determine how support can best be offered [51].

### Strengths and limitations

The great strength of this study lies in the in-depth understanding gained by consistent and respectful listening to people with MJD and their families, facilitated through participation of CRPs who share the language and cultural background of participants. Although the sample size was relatively small, it is important to recognise that participants shared an enormous breadth of experience arising from the impact of MJD on their families for generations. Repeated engagement between researchers and the small number of participants helped to build strong relationships that allowed sharing of stories in unprecedented ways. Motivation to tell their stories arose from participants’ belief that by sharing what they had learned, they could improve services for their families and others with MJD around the world. While not directly generalisable to other populations with MJD, this study offers important insights that families and service providers in other settings can draw on. With few previous studies on the experience of hereditary ataxia [52–54] and none specific to mobility, the findings of this current study contribute to emerging evidence about the need and priorities for rehabilitation in this population [48].

### Clinical and research implications

The findings of this study inform development of services responsive to the needs of individuals and families with MJD. The staying strong framework (Fig 2) can be used flexibly by services to identify the priorities of each individual with MJD and their family and accordingly, be used in the design of a program to keep them walking and moving around as long as possible. Use of the framework will ensure that the nature of supports provided will simultaneously promote physical, social, emotional and spiritual well-being. To determine feasibility of this approach, the next step is to pilot an intervention program to promote mobility that is based on the staying strong framework. From there, future research will be needed to build the evidence base to support this approach to secure sustained and sufficient investment in ongoing services tailored to the needs of individuals and families with MJD.

## Conclusion

Individuals and families with MJD from the Groote Eylandt Archipelago value walking and moving around because it enables them to do what matters most in life. To maintain mobility for as long as possible, they engage in lifestyle practices to strengthen their physical and psychosocial well-being. To support individuals and families with MJD, person-centred, holistic and strength-based services are required.
